# Novel Program Scheme of Vertical NAND Flash Memory for Reduction of Z-Interference

**DOI:** 10.3390/mi12050584

**Published:** 2021-05-20

**Authors:** Su-in Yi, Jungsik Kim

**Affiliations:** 1 Department of Electrical and Computer Engineering, Texas A&M University, College Station, TX 77843, USA; yisuin@tamu.edu; 2 Samsung Electronics, Hwasung 18448, Kyeonggi, Korea; 3 Department of Electrical Engineering, Gyeongsang National University, Jinju 52828, Gyeongnam, Korea; 4 Engineering Research Institute (ERI), Gyeongsang National University, Jinju 52828, Gyeongnam, Korea

**Keywords:** NAND flash memory, interference, Technology Computer Aided Design (TCAD) simulation, disturbance, program, non-volatile memory (NVM)

## Abstract

Minimizing the variation in threshold voltage (*V_t_*) of programmed cells is required to the extreme level for realizing multi-level-cells; as many as even 5 bits per cell recently. In this work, a recent program scheme to write the cells from the top, for instance the 170th layer, to the bottom, the 1st layer, (T-B scheme) in vertical NAND (VNAND) Flash Memory, is investigated to minimize *V_t_* variation by reducing Z-interference. With the aid of Technology Computer Aided Design (TCAD) the Z-Interference for T-B (84 mV) is found to be better than B-T (105 mV). Moreover, under scaled cell dimensions (e.g., *L_g_*: 31→24 nm), the improvement becomes protruding (T-B: 126 mV and B-T: 162 mV), emphasizing the significance of the T-B program scheme for the next generation VNAND products with the higher bit density.

## 1. Introduction

Due to the nature of NAND flash memory, which lacks the capability of random access [1] of NOR flash memory [2,3] or other memories such as DRAM (Dynamic Random Access Memory) and PCM (Phase Change Memory), reading and writing operations of one cell inevitably accompanies operations on the other cells simultaneously in a target NAND string [4,5]. Various combinations of the operation scheme such as bit line voltage (***V_BL_***), read voltage (***V_READ_***), pass voltage (***V_PASS_***), etc., are typically tested and finally the optimal set is chosen by product engineers to minimize the threshold voltage (***V_t_***) variation for the given as-fab-out chips [6,7,8,9]. Moreover, with the higher level of layers emerging every year or two, such that Memory companies announced a 6th generation vertical NAND (VNAND) flash memory product of 120 layers in 2019 and subsequently plan to announce the next 7th generation of 170 or more layers in a year or so [10], even more complicated combinations of the operation scheme are being developed. For example, varying bias conditions depending on the word line (WL) number, due to the nature of high aspect ratio contact etching [11,12,13], need to be investigated by trial and error to meet the criteria of ***V_t_*** variation in a tight schedule. For this reason, the operation scheme optimization process heavily relies on the product engineers’ intuition or, recently, statistical approaches such as machine learning technology which can often neglect to understand the underlying charge transport physics [14,15]. However, in order to accumulate the prior experience on the operation scheme optimization toward the sustainable technique for future products, it is critically important to understand the correlation between the input (operation scheme) and the output (***V_t_*** variation).

## 2. Simulation Methods

**Figure 1:**
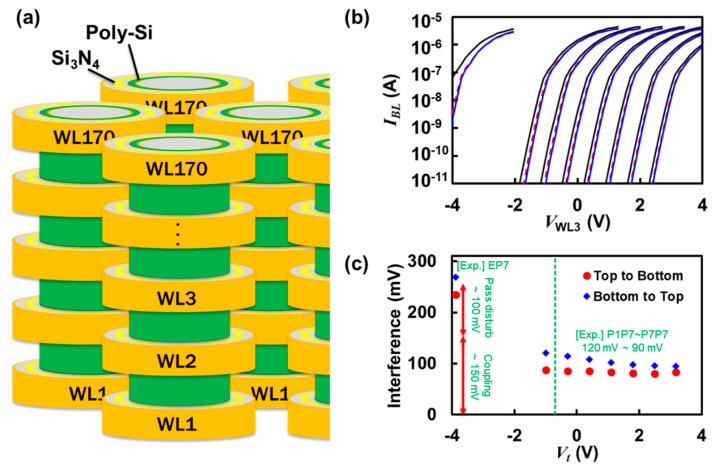
(**a**) Vertical NAND (VNAND) Flash cell array schematic showing neighbors both intra-string (Z-interference) and inter-string. Z-interference is the most critical since the channel is shared in close proximity for VNAND products. (**b**) Current versus voltage data as a function of the gate voltage of the victim cell (WL3). Solid line represents the reference state before the aggressor cell is programmed. Red dashed line and blue solid line denote the states after interference by T-B and B-T, respectively. (**c**) Interference of 8 different states (E, P1, P2, P3, P4, P5, P6, and P7) for triple level cell (TLC) under the condition of the aggressor programmed to P7 (*V_t_* = 3.177 V). Blue diamonds and red circles show the results out of Bottom to Top (B-T, WL4 is aggressor) and Top to Bottom (T-B, WL2 is aggressor), respectively. Remarks with (Exp.) denote experimentally measured interferences (unpublished) from Samsung’s 4th generation VNAND (Ref. [18]).

In this report, we target the investigation of ***V_t_*** interference and coupling dependency on the programming direction in a bit line as shown in Figure 1a. One method is to program beginning from the bottom to top (B-T), i.e., from WL1 to WL170, which is the scheme adopted by early generations of VNAND, and the other is to program beginning from the top to bottom (T-B), i.e., from WL170 to WL1, which has been recently employed [16,17,18,19,20,21]. Although the scheme of T-B is currently prevailing over B-T because of the better vulnerability toward interference/coupling, as mentioned earlier, this link may have been found through empirical trials based on a few prior reports with outdated cell geometries [22,23]. That is probably the reason why any quantitative analysis and investigation is unavailable publicly with up-to-date VNAND cell structures. In this work, by performing Technology-Computer Aided Design (TCAD) simulations (Synopsys^TM^, Mountain View, CA, USA) of interference for the two distinct schemes [24], we provide solid understanding on the difference between the two and evaluate the benefit for the scaled-down cells of next generation VNAND products.

## 3. Results and Discussion

Figure 1b shows eight sets of ***I_BL_***-***V_WL_*** curves at ***V_t_***’s from the erased state (E) to the programmed states (P1, P2, …, P6, and P7). The tunneling masses of 0.36 *m*_0_ and 0.38 *m*_0_ were used for electrons and holes, and the block erasing with ***V_ERS_*** = −16 V for 1 ms resulted in ***V_t.E_*** = −3.889 V based on BL current ***I_BL_*** = 100 nA. It should be noted that the electron tunneling mass of 0.36 *m*_0_ was chosen to properly describe the pass disturb under EP7 interference (approximately 100 mV of pass disturb and 150 mV of coupling), while this brings about the programming speed faster (*V*pgm = 16 V for 100 μs makes ***V_t,P7_***) compared to experimental results (*V*pgm = 19~20 V for 100 μs makes ***V_t,P7_***). This is a well-known dilemma for Flash memory TCAD simulations, where the trap-assisted-tunneling (TAT) model is rarely considered due to the complexity in describing the atom defects in the actually fabricated ONO (Oxide-Nitride-Oxide) films. Moreover, uncertainties due to random telegraph noise (RTN) were not considered to clarify the comparison by mean *V_t_*’s [25]. Read voltage (***V_READ_***) of 7 V was used as default. Once every cell in the model (five word lines) was written to the state E by the block erasing, seven different programmed states were mimicked by using the programming voltage (***V_PGM_***) of 16.0 V, 15.3 V, 14.6 V, 13.9 V, 13.2 V, 12.5 V, and 11.8 V for P7, P6, P5, P4, P3, P2, and P1 states, respectively, on the third word line (WL3) together with ***V_PASS_*** applied to the other cells of 8 V for 100 μs. Consequently, the ***V_t_***’s of seven programmed states constituted 3.177 V, 2.487 V, 1.794 V, 1.098 V, 0.399 V, −0.293 V, −0.990 V, of which the average read window between two adjacent states is approximately 0.7 V, enabling the triple level cell (TLC). The interference on WL3 was simulated under two different scenarios. The first is when the upper adjacent cell, WL4, is programmed to P7, named as B-T scheme and represented by blue solid lines. The second is when the aggressor is WL2, named as T-B scheme and represented by red dashed lines.

Based on the raw data available in Figure 1b, the amount of interference in the unit of mV as a function programmed state in ***V_t_*** is rearranged in Figure 1c, which can be labelled as EP7, P1P7, P2P7, P3P7, P4P7, P5P7, P6P7, and P7P7. The green dashed line at ***V_t_*** = −0.690 V is of the virgin state and it should be noted that the fixed charge of −10^12^ cm^−2^ was used between the poly-silicon channel and fill oxide (the core oxide of a NAND string due to macaroni-like structure) to fit the typical virgin ***V_t_*** ranging from −0.5 V to +0.5 V. The comparison between T-B (red circles) and B-T (blue diamonds) clearly provides the better interference performance of T-B over B-T. The interference in NAND Flash consists of two contributions: one is the change in trapped charge concentration of the victim cell due to ***V_PASS_*** = 8 V during the programming phase (pass disturb), and the other is the influence of the adjacent cell during the reading phase (coupling). In addition, the distinctively high interference of EP7, 269 mV for B-T and 235 mV for T-B, compared to those of P1P7~P7P7 implies the significant contribution of pass disturb.

**Figure 2:**
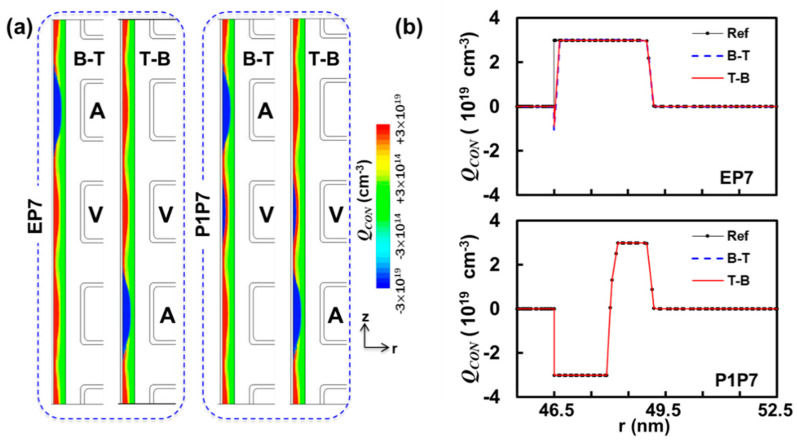
(**a**) Net charge concentration (***Q_CON_***) comparison of B-T and T-B. (**b**) ***Q_CON_*** in the trap-nitride layer of WL3 as a function radial coordinate r, where 46.5 nm and 52.5 nm represent two interfaces with oxide layers: top, EP7, where WL3 is originally at the state with *V_t_* = −3.889 V (E). Slight change in ***Q_CON_*** for 46.5 < r (nm) < 46.7 is observed after interference, because of pass disturb (8 V); bottom, P1P7, where WL3 is initially at the state with *V_t_* = −0.976 V (P1). In this case, pass disturb is negligible because P1 state is relatively invulnerable to ***V_PASS_*** = 8 V.

Figure 2 provides the net charge concentration (***Q_CON_***) information with color plots (a) and curves (b) as a function of the position in the radial axis. Note that r = 0 nm corresponds to the center of the cylindrical symmetry for a VNAND string. Because the diameter of the hole was used to be 120 nm followed by 7.5 nm blocking oxide, 6 nm trap-nitride, and 5.5 nm tunneling oxide, *r* = 46.5 nm and *r* = 52.5 nm represent the interfaces with tunneling oxide and blocking oxide, respectively. In this work, we did not consider the tendency of decreasing hole-diameters and ONO film thicknesses with decreasing WL numbers in so called “stack-coverage”. Although it is known to cause the variation in threshold voltages of 3D NAND cells [26,27], recent advances in high-aspect-ratio thin film technique produce very decent stack-coverages (ONO film > 95% and Poly-Si > 90% by comparing the film thickness of WL1 to that of WL170). Moreover, the state-of-the-art high-aspect-ratio-etching technique makes almost uniform hole diameters (~120 nm) except for approximately 10% of the top and bottom layers of a NAND string [21].

The first two color plots in Figure 2a show the comparison between B-T, where WL4 was programmed to P7, and T-B, where WL2 was programmed to P7, so that all other cells appear to be similar with the peak net charge concentration of 3 × 10^19^ cm^−3^ except for aggressor cells with −3 × 10^19^ cm^−3^. Note that the trap concentration, both for electrons and holes, was set to 3 × 10^19^ cm^−3^ in this work. Even though only five WLs were built in our simulation model, considering the computational cost, there was no detectable amount of asymmetry between the cell near the top (WL4) and the cell near the bottom (WL2) in terms of the net charge concentration. The plot of ***Q_CON_*** as a function of *r* in Figure 2b reveals the subtle change in the net charge concentration after the aggressor cell (WL4 or WL2) is written, especially near the interface of trap-nitride and tunneling oxide (46.5 < *r* (nm) < 46.7). The integration of the net charge concentration, with respect to the volume, led to Δ***Q*** about −20, where B-T and T-B showed negligible difference. The color plots for P1P7 on the right in Figure 2a show P1P7 interference where the victim is programmed to P1 before the aggressor is written to P7, so that slight blue color region (***Q_CON_*** < 0) is identified together with the trapped holes from the block erasing operation. The plot of ***Q_CON_*** versus r in Figure 2b for P1P7 demonstrates the coexistence of trapped electrons near the interface with tunneling oxide (46.5 < *r* (nm) < 48) and trapped holes farther away from the interface (48 < *r* (nm) < 49). More importantly, all three curves (Ref, B-T, and T-B) are almost overlapped and the corresponding integration of the difference concluded that the charge equivalent to just one electron tunneled through the victim cell under ***V_PASS_*** = 8 V for 100 μs. In order to explain the sudden jump in interference from P1 to E, the information of the change in the net charge can be utilized. The upper bound of the ***V_t_*** shift, as a result of the additional 20 trapped electrons, can be estimated by Δ***V_t_*** = 1.6 × 10^−19^ × Δ***Q***/***C*** with the assumption of a simple one-dimensional capacitor. ***C*** was calculated to be 20.6 aF by ***C***^−1^ = ***C***_TOX_^−1^ + ***C***_TrapN_^−1^ + ***C***_BOX_^−1^, and results in Δ***V_t_*** = 155 mV by Δ***Q*** = 3.28 aC (20 electrons), whereas for P1P7 interference the contribution of Δ***Q*** to interference is just 8 mV because only one electron was additionally trapped. Therefore, the distinctively high interference for EP7 should be attributed to the tunneling under ***V_PASS_*** = 8 V for 100 μs, whereas P1P7 allows negligible tunneling under the same condition.

**Figure 3:**
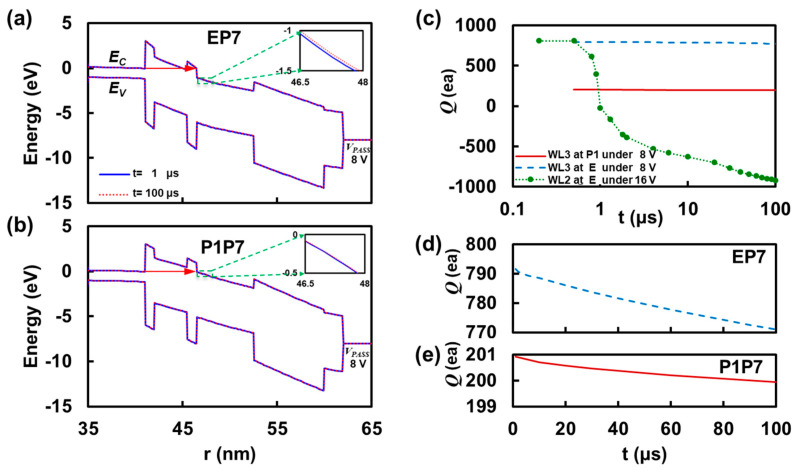
Band diagram of the victim cell (WL3) along the radial direction of a cylindrical cell string and corresponding number of trapped charges in the trap-nitride layer as a function of time while programming WL2 with ***V_PGM_*** = 16 V and ***V_PASS_*** = 8 V. (**a**) WL3 at the state E exhibits Fowler–Nordheim tunneling due to lowered conduction band edge by trapped hole charges in the trap-nitride layer. (**b**) WL3 at the state P1 depicts the harsher tunneling barrier compared to that of E in Figure 4a. This is because the net charge in the trap-nitride layer is less positive compared to E(erase) state so that the electrostatic potential is higher. (**c**) Number of trapped charges (***Q***) in the trap-nitride layer of WL2 beginning from the state E as a function of time under programming voltage ***V_PGM_*** = 16 V is shown (green dotted line) together with that of victim cell under two different states (E and P1). (**d**) WL3 at E under the bias ***V_PASS_*** = 8 V shows the charge in ***Q*** from +792 to +771, implying about 20 electrons were tunneled and holes were canceled. (**e**) WL3 at P1 shows negligible change in ***Q*** (from +201 to +200) so that the interference (121 mV for B-T and 88 mV for T-B) purely comes from the adjacent cell’s channel inversion.

Figure 3a,b shows the band diagram for WL3 along with the radial direction from r = 35 nm (interface between poly-silicon channel and fill-oxide) to *r* = 65 nm (tungsten gate) for the aforementioned cases, EP7 and P1P7. Due to the lower conduction band edge (or electrostatic potential) of the trap-nitride layer stemming from the trapped hole (792 holes trapped after block erasing shown in Figure 3c), the tunneling barrier from the conduction band edge of the channel is partially Fowler–Nordheim type. As a result, the conduction band edge’s up-lift of about 0.03 eV can be observed at *t* = 100 μs, compared to *t* = 1 μs on the inset. However, P1P7 in Figure 3b exhibits a harsher tunneling barrier because P1 state possesses only 201 holes as shown in Figure 3e; hence, the electrostatic potential of Si_3_N_4_ is relatively higher than that of the state E. The inset shows negligible change in the conduction band edge during 100 μs, which is consistent to the statement for P1P7 of Figure 2b (only 1 electron tunneled). Figure 3c shows the change in the number of net charges in the trap-nitride layer of WL3 as a function of time. The aggressor under 16 V shows nonlinearly fast electron tunneling as a function time, where 807 holes initially located in WL2’s trap-layer are almost cancelled to neutral within 1 μs and, for the rest of the time, the additional charge corresponding to 918 electrons is trapped until *t* = 100 μs. Figure 3d,e show the change with time for EP7 and P1P7, respectively. Because the range of change is significantly small (EP7: 20 electrons and P1P7: 1 electron) compared to the aggressor cell at a larger bias of 16 V, the time-dependent evolution appears to be simple linear evolutions.

**Figure 4:**
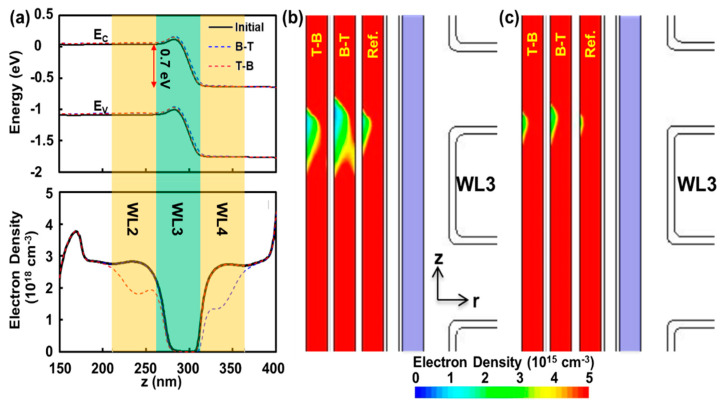
Poly-silicon channel information during reading operation (*V_WL3_* = −1 V, ***V_READ_*** = 7 V, ***V_BL_*** = 0.7 V, ***V_CSL_*** = 0 V) (**a**) band diagram: Top, electron carrier concentration; bottom, following the z-axis (r = 38 nm). The potential of 0.7 V through the bit line is mainly applied to the reading cell (WL3) since the adjacent cells are fully inverted with ***V_READ_*** = 7 V; hence they have negligible resistances. Consequently, WL4 should experience less inversion (by ***V_READ_*** − 0.7 V = 6.3 V) compared to WL2 (by ***V_READ_*** − 0 V = 7 V), which is reflected in electron density in the bottom figure. WL4 and WL2 have carriers of 1.3 and 1.9 (10^18^ cm^−3^) at the center, respectively. (**b**) Color plots of electron density for ‘initial’ reveal non-centered carrier bottleneck due to drain-induced-barrier-lowering (DIBL) effect. As a result, B-T; having the upper adjacent cell programmed, has the stronger interference compared to T-B with the lower adjacent cell programmed. The light blue region corresponds to trap-nitride layer (Si_3_N_4_) (**c**) When ***V_READ_*** is increased to 8 V, the imbalance between B-T (***V_READ_*** − 0.7 V = 7.3 V) and T-B (***V_READ_*** − 0 = 8 V) is reduced.

Now, P1P7 can be regarded as the best example to investigate the mechanism of improved interference performance for T-B over B-T because it allows us to rule out Δ***Q*** even after experiencing ***V_PASS_*** = 8 V for 100 μs (pass disturb), whereas the contrast is the largest among others: P2P7, P3P7, …, P7P7. Figure 4a shows the P1P7 case’s band diagram for poly-silicon channel through the axial direction z when ***V*_WL3_** = −1 V, which is approximately the ***V_t_*** of P1 state (−0.99 V), is being applied on WL3 and ***V_READ_*** = 7 V for the other cells. Due to the partial inversion of WL3 with −1 V compared to WL2 and WL4 with 7 V, the voltage applied to BL (***V_BL_*** = 0.7 V) is mainly applied to solely WL3. As a result, the upper cells, including WL4, should encounter drain-induced-barrier-lowering (DIBL), hence the actual potential drop across ONO should be 6.3 V (***V_READ_*** − ***V_BL_***). The plot at the bottom of Figure 4a reveals the electron carrier density, which shows the slightly lower carrier concentration for the WL4 region compared to that for the WL2 region. Moreover, the minimum carrier concentration 5.1 × 10^15^ cm^−3^ appeared at *z* = 303 nm, which is above the center of WL3 (*z* = 287.5 nm) and reflects the effect of DIBL. Figure 4b visualizes the off-centered ‘bottleneck’ for conduction. It should be noted that the red-colored region represents that the carrier density is equal to or higher than 10^15^ cm^−3^. Due to the off-centered bottleneck based on DIBL, the aggressor on the upper adjacent cell (WL4 for B-T case) strengthens the bottleneck which reflects high interference (121 mV in Figure 1c). For T-B case, the bottleneck is less affected by the aggressor at the lower cell (WL2) so that the interference is reduced significantly (88 mV in Figure 1c). Figure 4c shows a similar comparison under higher read voltage, ***V_READ_*** = 8 V. Considering that the contrast between T-B and B-T comes from the DIBL effect on ***V_READ_***, it is observed that the higher reading voltage lessens the difference between the two.

It is worth inspecting the trend of T-B compared to B-T under various circumstances and scaled cell dimensions that are inevitable for the next generation of products with more layers; unless semiconductor process hurdles related to vertical NAND’s stack height are dramatically resolved, such as high aspect ratio etching technique and mechanical stress issues, to name a few [28].

**Figure 5:**
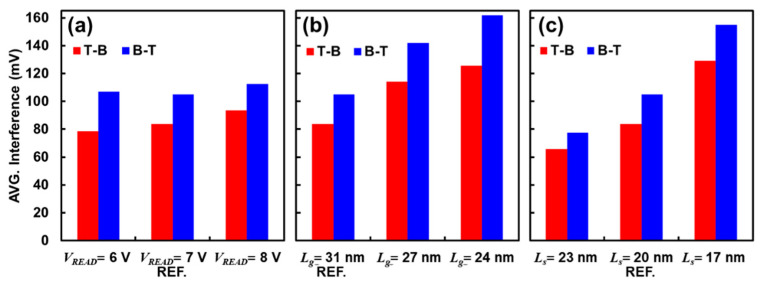
Averaged interference (P1P7, P2P7, …, P6P7, and P7P7) for T-B and B-T schemes with various changes such as (**a**) ***V_READ,_*** and cell dimensions, (**b**) gate length and (**c**) gate space, for the next generation vertical NAND Flash products. Note that the reference is (***V_READ_***, *L_g_*, *L_s_*) = (7 V, 31 nm, 20 nm) and the raw data of each case is available in Figure S1.

Figure 5a shows the variation with respect to read voltage difference. It can be seen that the improvement by T-B over B-T is protruding with smaller read voltage such that ***V_READ_*** = 6 V shows the improvement of 28 mV (= 107 mV − 79 mV), whereas ***V_READ_*** = 8 V exhibits 19 mV (= 113 mV − 94 mV) when considering the averaged value of P1P7, P2P7, …, P6P7, and P7P7. Figure 5b,c depict the trend with scaled dimensions where 24 nm for the thickness of the nitride pad during the initial stage of the VNAND process (*L_g_*) and 17 nm for the thickness of the oxide pad (*L_s_*) are highly probable for the newest vertical NAND Flash Memory product (>170 layers) under development. It is clearly shown that the scaled cells undergo significant interference such that *L_g_* = 24 nm shows 162 mV and *L_s_* = 17 nm shows 155 mV under B-T. Luckily the remedy by T-B over B-T also increases with scaling such that *L_g_*/*L_s_* = 24 nm/20 nm shows the improvement of 36 mV, which is superior than 21 mV from the reference geometry of this work (*L_g_*/*L_s_* = 31 nm/20 nm) so that the deterioration in interference and read window can be slowed down. It is noted that we simulated thicker ONO and Poly-Si cases (7.8/6.3/5.8/6.6 nm) compared to the reference (7.5/6.0/5.5/6.0 nm) to confirm any remarkable deviation owing to the stack-coverage. Nevertheless, the interference for T-B and B-T were found to be 114 mV and 86 mV, respectively, such as the reference of 105 mV for T-B and 84 mV for B-T. Therefore, we believe that the state-of-the-art stack coverage (ONO > 95% and Poly-Si > 90%) in the Flash memory product’s thin-film process is sufficiently good enough to impose little uncertainties in our simplified TCAD models.

**Figure 6:**
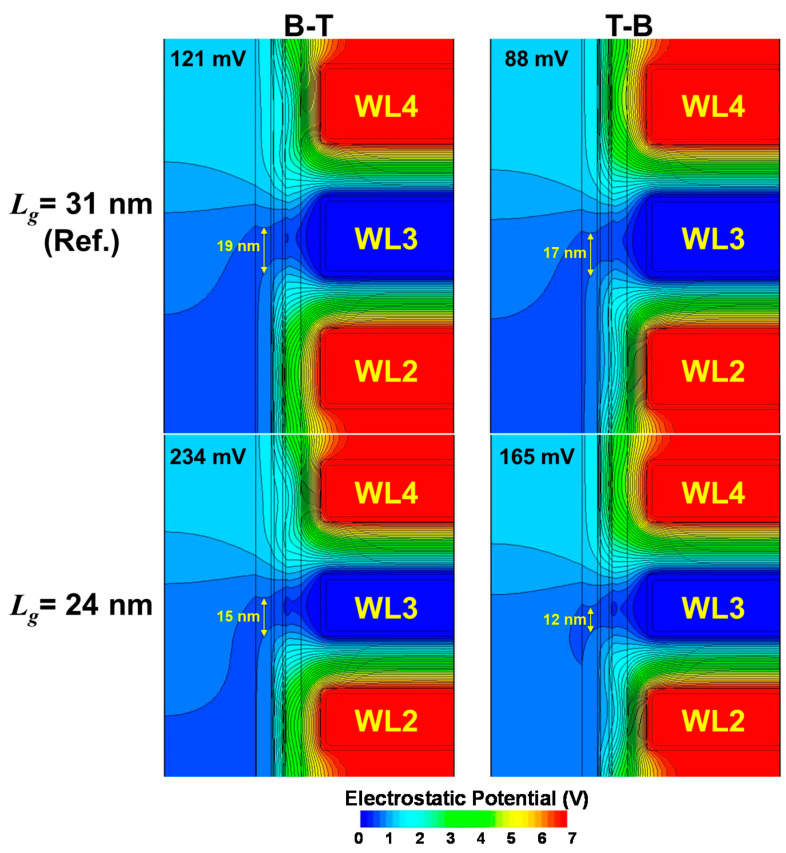
Electrostatic potential distribution change with *L_g_* scaling (31 → 24 nm) Both are after EP7 interference (***V_t,Victim_*** = −0.99 V, ***V_t,Aggressor_*** = 3.2 V) followed by reading at the moment at ***V*_WL3_** = −1 V.

Figure 6 exposes the corresponding electrostatic potential distribution for *L_g_* = 24 nm compared to the reference *L_g_* = 31 nm, further analyzing the improvement by T-B scheme for scaled cells as an example. It should be noted that the electrostatic potential is referenced to that of WL3. P1P7 interference, where ***V_t,_*_WL3_** = −0.99 V and ***V_t,_*_Aggressor_** = 3.18 V, was used consistently for the analysis in Figure 4, which exhibits the improvement from 121 mV of B-T to 88 mV of T-B as shown in Figure 1c. Note that the case of *L_g_* = 24 nm makes 234 mV from B-T and 165 mV from T-B, which is higher than the averaged values available in Figure 5. The electrostatic potential valley is mainly responsible for the ***V_t_*** of the cell under reading, and it is observed that at the center of the channel (*r* = 38 nm) the length of the valley (0.4 V < electrostatic potential < 0.47 V) changes dramatically for the scaled cell (15 nm → 12 nm at *L_g_* = 24 nm), compared to the reference (19 nm → 17 nm at *L_g_* = 31 nm). The emphasized deterioration in interference with scaled NAND cell sizes is indirectly evidenced by a 14 nm planar NAND flash memory reported in 2016 by Samsung [23]. Although they did not adopt the scheme of T-B [22] and kept the conventional B-T due to undisclosed reasons, a significant interference (back pattern dependency or back-pattern-effect) in the extremely scaled 14 nm NAND cells might have forced them to introduce a new scheme in incremental step pulse programming (ISPP), where ***V_READ_*** is lowered selectively for upper cells during the verify operation in ISPP.

## 4. Conclusions

In conclusion, this work performed a systematic study on the improvement in interference when the Top to Bottom (T-B) programming scheme is employed compared to the conventional Bottom to Top (B-T) scheme which probably originated from the planar NAND Flash products with a single layer on the ground level in a historical point of view. With the aid of TCAD simulations, it is shown that only the erased state (E) suffers from both pass disturb under the normal condition of ***V_PASS_*** = 8 V and coupling to the adjacent cells. The enhancement by the T-B scheme is mainly delivered by the latter contribution (coupling), stemming from the nature of NAND’s reading operation combined with drain-induced-barrier-lowering (DIBL). Therefore, most states (e.g., P1, P2, …, P6, P7 for TLC and P1, P2, …, P14, P15 for QLC) can benefit from the T-B scheme, despite the fact that programmed states are inherently free from pass disturb. Moreover, it is expected that T-B lessens the interference more prominently, especially for the next generation vertical NAND Flash products with more than 170 layers, inevitably followed by the higher degree of integration (smaller *L_g_* and *L_s_*). This work highlights its importance for future vertical NAND Flash memories, the applications of which include conventional use as data storage [21], but also other applications such as neuromorphic computing [29,30,31,32], security in IoTs [33], etc.

## Supplementary Materials

The supporting materials are available online at https://www.mdpi.com/article/10.3390/mi12050584/s1. Figure S1: Raw data of interference under various conditions of *V_READ_*, *L_g_*, and *L_s_* with respect to the reference *V_READ_* = 7 V, *L_g_* = 31 nm, and *L_s_* = 20 nm shown in Figure 5.
