# Supplementary material for: Novel Program Scheme of Vertical NAND Flash Memory for Reduction of Z-Interference
Source: Micromachines (Basel). 2021 May 20;12(5):584. doi: 10.3390/mi12050584 (PMC8160891; doi:10.3390/mi12050584)
Supplement: Supplementary file 1 [file micromachines-12-00584-s001.zip › micromachines-1203753-supplementary.pdf]

Supplementary Materials

Novel Program Scheme of Vertical NAND Flash Memory for Reduction of Z-Interference

Su-in Yi, and Jungsik Kim

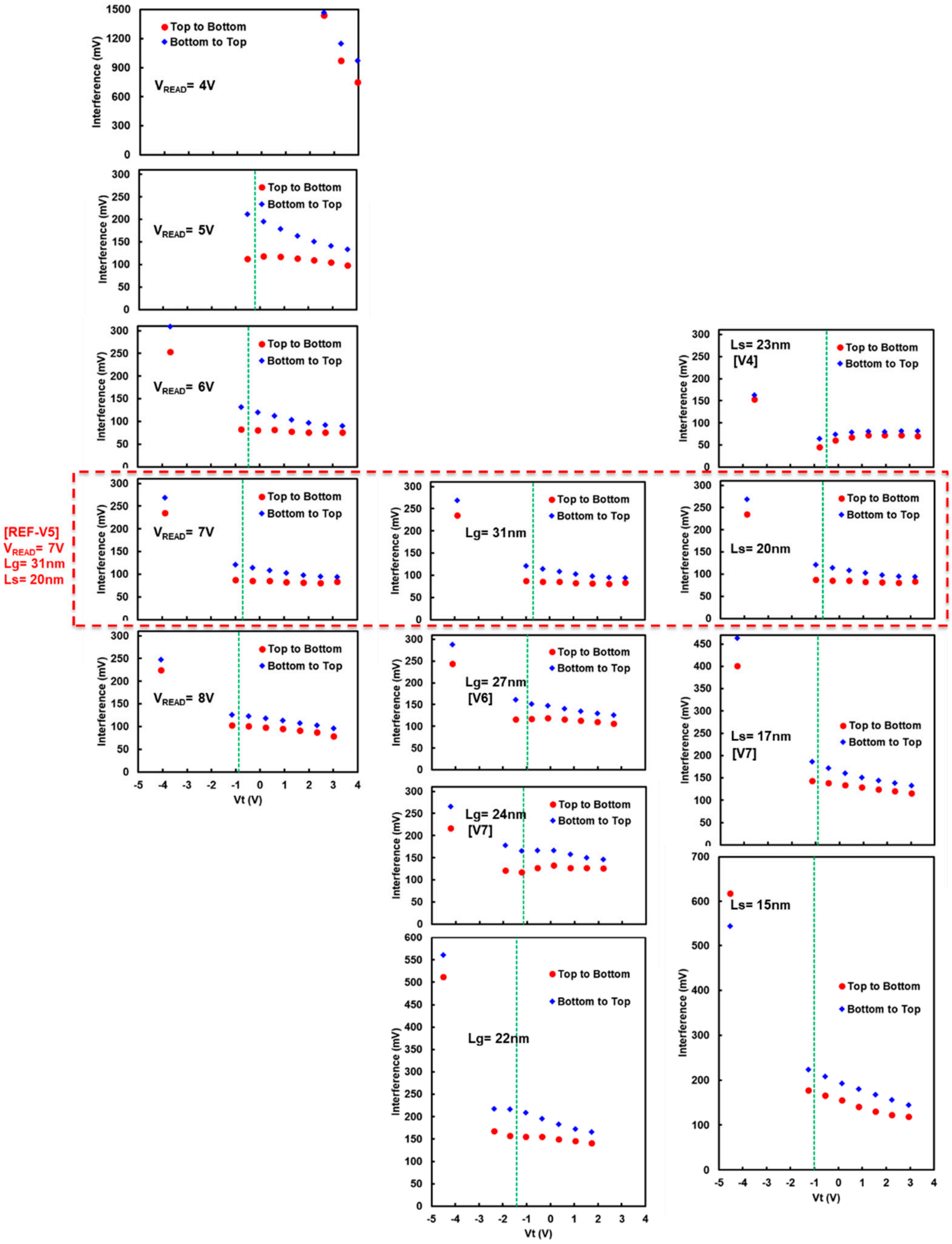

Figure S1. Raw data of interference under various conditions of V_{READ}, L_g, and L_s with respect to the reference V_{READ}= 7 V, L_g= 31 nm, and L_s= 20 nm shown in Figure 5.
